# Screening for Non-polyenic Antifungal Produced by Actinobacteria from Moroccan Habitats: Assessment of Antimycin A19 Production by *Streptomyces albidoflavus* AS25

**DOI:** 10.22088/IJMCM.BUMS.7.2.133

**Published:** 2018-06-03

**Authors:** Ahmed Nafis, Najoua Elhidar, Brahim Oubaha, Salah Eddine Samri, Timo Niedermeyer, Yedir Ouhdouch, Lahcen Hassani, Mustapha Barakate

**Affiliations:** 1 *Laboratory of Biology and Biotechnology of Microorganisms, Faculty of Sciences Semlalia, Cadi Ayyad University, Marrakech, Morocco.*; 2 *Interfaculty Institute of Microbiology and Infection Medicine (IMIT), Eberhard Karls University Tübingen, Tübingen, Germany.*; 3 *Department of Biology, Nador Multidisciplinary Faculty, Mohamed First University, Nador, Morocco.*; 4 *Institute of Pharmacy, Pharmaceutical Biology, Martin-Luther-University Halle-Wittenberg, 06120 Halle (Saale), Germany.*

**Keywords:** Non-polyenic antifungal compounds, Actinomycetes, antimycin A19

## Abstract

Fungal diseases are currently a serious public health problem, due to the limited number of fact-based effective principles, and the emergence of resistant strains to the polyenic antifungals. The aim of this study was to screen, for non-polyenic antifungals production by Actinobacteria, and to validate the screening program by characterizingthe produced compounds.Actinobacteria isolates were tested against four clinic human-pathogenic fungi isolated from Hospital Mohammed V Rabat, Morocco. The production of non-polyenic antifungal metabolites by active isolates was investigated based on the yeast cell specificity as challenging targets, antibacterial activity, activity against resistant *Candida tropicalis* R2 and *Pythium irregular* (resistant to polyenes), inhibition of antifungal activity by the addition of exogenous ergosterol, and the UV-visible light spectrophotometric analysis of the active crude extracts.The antifungal compound produced was purified using various chromatographic techniques and the selected producing strain was identified using the polyphasic approach.Among 480 Actinobacteria isolates, 55 showed antifungal activity against all tested clinically derived fungi. After performing the screening program, 4 Actinobacteria that hadall the desired criteriawere selected. Using the polyphasic approach, the taxonomic position of the selected Streptomyces AS25, isolated from rhizospheric soil of *Alyssum spinosum*, showed that it belongs to Streptomyces genus with 100% partial 16S similarity with *Streptomyces albidoflavus* NBRC13010. On the basis of HPLC and mass spectrometry, the purified compound produced by Streptomyces AS25 was identified as a non-polyenic lactone, antimycin A19, which has been found for the first time to be produced by *Streptomyces albidoflavus* strain. Following the obtained results, it is important to note that our screening criteria for non-polyenic antifungals have been validated and the rhizospheric soil represents an interesting source to isolate Actinobacteria.

In recent years, fungal infections have been gaining prime importance because of the morbidity of hospitalized patients (1). In particular, dermatophytosis and aspergillosis have remained the opportunist fungal infections that occur most frequently. They are a group of closely related filamentous fungi that have the ability to invade keratinized tissues such as skin, hair, and nail of humans and animals to produce an infection (2). These fungal infections are caused essentially by *Trichophyton*, *Microsporum*, and *Aspergillus-*species for dermatophytose and aspergillosis diseases, respectively (3).

Despite the high number of antibiotics available in the market, antifungal antibiotics are a very small but significant group of drugs, and have an important role in the control of infectious diseases. Only a limited number of antifungal agents are currently available for the treatment of life-threatening fungal infections (4).

The antifungals in therapy present an important toxicity due to the biochemical similarity between fungal pathogens and infected host (all eukaryotes), and the increase of mycosis caused by opportunistic fungi encourages the research for novel antifungal antibiotics (5). However, many compounds such as polyenes in particular, cannot be used because of their toxicity, while they are of interest in chemotherapy. These antifungal agents show some limitations, such as significant nephrotoxicity of amphotericin B (6) and emerging resistance to the azoles (7). Lipid formulations of polyenes with lower toxicity and new triazoles (voriconazole and pasaconazole) with a wider spectrum of action (despite several recent improvements), including activity against some azole-resistant isolates (8) guide a way out of this restraint.

The search for a new, safer, broad – spectrum antifungal antibiotic with greater potency has been progressing slowly (9). The development of new antifungal agents, preferably naturally occurring with novel mechanisms of action, is an urgent medical need and the fungal cell is a promising drug target for antifungal therapy.

Microbial natural products have been one of the major sources to discover novel antifungals, and the chance of isolating new metabolites are low unless adequate isolation strategies are adopted.

Among the microorganisms, Actinobacteria are the most economically and biotechnologically useful prokaryotes(10). They produce antibiotics and other industrially important secondary metabolites (11,12). Approximately 70% of all known antibiotics were isolated from actinomycetes, amongwhich 75% were employed in medicine and 60% in agriculture (13).

The aim of the presentstudy wasto screen the non-polyenic antifungal compounds produced by Actinobacteria isolated from different Moroccan habitats, and to validate non-polyenic screening criteria by the identification of the producing strain and elucidation of the structure of elaborated-compounds.

## Materials and methods

**Screening of actinomycetes for antifungal and antibacterial activity**

The antifungal activity was tested by agar diffusion assay using 480 actinomycete isolates originating from various Moroccan ecosystems, such as rhizospherical soil, endophytic plants, Atlas Mountain soil, and Sahara sand.

The isolated strains were spread over the entire surface of Bennett medium Petri dishes (14). As soon as the microorganism developed, agar discs were cut out using a cork borer (6 mm diameter), and were transferred to the surface of Sabouraud dextrose agar plates seeded with five clinically mold species obtained from the Moroccan Coordinated Collections of Micro-organisms (CCMM) in the National Center for Scientific and Technical Research Morocco (CNRST) (Table 1).

**Table 1:** Origin of the clinical yeast strains used for microorganisms test

Mold strain		Origin		Access number	
*Aspergillus parasiticus*		Clinic		CCMM-M24	
*Aspergillus niger*		Human ear		CCMM-M100	
*Scorpulariopsis candida*		Human toenail		CCMM-M84	
*Microsporum canis*		Human arm		CCMM-M103	
*Trichophyton rubrum*		Human big toenail		CCMM-M127	

Plates were kept at 4°C for 2 h, then incubated at 30°C for filamentous fungi to allow the growth of test organisms, and examined for antibiosis after 24 to 48 h. If the antibiotic produced by the organism inhibited the growth of the test organism, a clear zone was formed around the discs(15).

**Screening for antifungal non- polyenic meta-bolites**

The selection of isolates producing only non-polyenic antifungal metabolites was carried out using four- test-criteria:

- The antibacterial activity was evaluated on Mueller Hinton agar plates, previously inoculated with the test microorganism (10^5^-10^6^ CFU/ml). The bacteria used as target were *Pseudomonas aeruginosa *DSM 50090*, Escherichia coli *ATCC 8739, *Staphylococcus aureus *209 PCIP 53156*, Micrococcus luteus *ATCC 381, and *Bacillus subtilis *ATCC 14579from the LBBM laboratory collection, Morocco. Plates were kept at 4 °C for 2-4 h and then incubated at 37 °C for 18–24 h. The zone of inhibition was were then measured (16).
- The study of activity against *Candida tropicalis R2 *DSM11953 and *Pythium irregular *(CIP18) (resistant against polyenes such as amphotericin B and nystatin).
- In order to determine the effect of the antifungal from selected active actinomycete isolates on the ergosterol present in the fungal cell membrane, ergosterol was tested for its ability to reverse the inhibition of the test microorganismsby the antifungal strain (17). Sabouraud’s agar plates with 50 mg/ml ergosterol were prepared along with a control without ergosterol. The plates were inoculated with *Aspergillus niger *M100. Ergosterol inhibition was tested by disc diffusion method. Sterile filter paper discs (6 mm in diameter) were impregnated with 30 µl ergosterol suspension, dried and placed onto plates previously seeded with test microorganisms. The plates were incubated at 28°C for 24 h and examined for zone of inhibition. The absence of change in inhibition diameter indicates the non-polyenic nature of the tested molecule.
- Crude antimicrobial extract was prepared from the culture filtrate of each active isolate by solvent extraction usingethyl acetate. Ethyl acetate was added to the filtrate with 1:1 (v/v) ratio, and shaken vigorously for 20 min. The organic phases were collected, and the organic solvent was removedusing a vacuum evaporator at 40°C to obtain a crude extract.

The absorption spectrum of active extracts in methanol as recorded in the UV region (210-400 nm) using a UV-visible spectrophotometer, and compared with those of known polyenic antifungal antibiotics (18).

**Characterization of selected isolates**

***Culture, morphological and physiological cha-racterristics***

The culturefeatures of the strains showing strong activity were characterized following the directions given by the International Streptomyces Project (ISP) media, namely: ISP1 agar (tryptone, 5 g; yeast extract, 3 g; agar, 20 g; H2O, 1000 ml, pH 7.2), ISP2 agar (yeast extract, 4 g; malt extract, 10 g; glucose, 4 g; agar, 20 g; H2O, 1000 ml, pH 7.2), ISP3 agar (meals, 20 g; MnCl2, 4H2O, 0.1 g; FeSO4, 7H2O, 0.1 g; ZnSO4, 7H2O, 0.1 g; agar, 18 g, H20, 1000 ml, pH 7.2), ISP4 agar (starch soluble, 10 g; K2HPO4, 1 g; MgSO4, 7H2O, 1 g; NaCl, 1 g; (NH4)2SO4, 2 g; CaCO3, 2 g; agar, 20 g; H20, 1000 ml, pH 7.2), ISP5 agar (L-asparagine, 1 g; glycerol, 10 g; K2HPO4, 1 g; MnCl2, 4H2O, 0.1 g; FeSO4, 7H2O, 0.1 g; ZnSO4, 7H2O, 0.1 g; agar, 20 g, H2O, 1000 ml, pH 7.2) at 28°C for seven to 14 days (19), and the Bergey’s Manual of Systematic Bacteriology (20).

The selected isolateswere tested for their ability to grow at pH 5 to 10 and at a temperature range from 25 to 45°C. Thus, actinomycete cultures were spot inoculated onto ISP2 agar plates featuring pH values of 5, 6, 7, 8, 9 and 10. The plates were checked for growth after seven days of incubation at 30°C. The same procedure was followed for the temperature test except for the use of ISP2 medium at pH 7.2. The temperatures tested were 27, 30, 37, 40 and 45°Crespectively (21).

Actinomycetes synthesize and excrete dark pigments, melanin or melanoid, which are considered to be a useful criterion for taxonomical studies (22). The production of melanoid pigments was carried out on ISP6 agar (peptone, 20 g; ferric citrate ammoniacal, 0.5 g; sodium thiosulfate, 0.08 g; yeast extract, 1 g; K2HPO4, 1 g; Agar 15 g; H2O, 1000 ml, pH 7.2) and ISP7 agar (glycerol, 15 g; L-tyrosin, 0.5 g; L-asparagine, 1 g; K2HPO4, 0.5 g; MgSO4, 7H2O, 0.5 g; NaCl, 0.5 g; FeSO4, 7H2O, 0.01 g; standard saline solution, 1 ml; agar, 18 g; H20, 1000 ml, pH 7.2) (23).

The ability of the isolates to utilize different carbon sources was determined on plates containing ISP basal medium 9 ((NH4)2SO4, 2.64g; KH2PO4, 2.38g; K2HPO4, 5.65g; MgSO4, 7H2O, 1g; standard saline solution, 1ml; agar, 15g; H20, 1000ml, pH 7.2) to which carbonsources were added to a final concentration of 1% (24). The plates were incubated at 28°C, and the growth was evaluated visually for little or no observable growth (+/-), moderate growth (++), or abundant growth (+++).

***DNA extraction, PCR amplification, and sequencing of 16S rDNA***

The selected isolate was cultivated for 4 days at 28°C in 50 ml of ISP2 medium under orbital shaking. Biomass was harvested by centrifugation at 8000 rpm for 10 min and washed twice with sterile distilled water. The genomic DNA of the pellet was extracted using PeqGold Bacterial DNA kit (Peqlab,Germany) according to the manufacturer as instructions. The purity of isolate DNA was checked and quantified according to standard procedures.

The 16S rDNA gene was amplified by polymerase chain reaction (PCR) using primers 27F bac (5’AGAGTTTGATCMTGGCTCAG3’) and 1492 Runi (5’ TACGGYTACCTTGTTACGACTT 3’) (25). PCR amplification was carried out using the thermocycler programmed as follows: a hot start of 95°C for 5 min, followed by 30 cycles of amplification at 95 °C for 1 min, annealing at 54-55°C for 1 min, extension at 72°C for 2 min, and finalextension at 72°C for 10 min. Finally, the tubes were held at 4°C for direct use, or stored at -20°C until needed.

The same primers were then used separately in two sequencing reactions from the two ends of the amplified fragment (about 1.5 kb). The two sequences obtained were compared for similarity with those contained in genomic database banks, using NCBI BLAST and EzTaxon (26). The phylogenetic tree was constructed using the neighbor-joining method with the Mega 6 software (27).

**Chemical characterization of active secondary metabolites**

***Fermentation and extraction of the active***

***compounds***

The selected isolate 1 (AS25) was grown on Bennett agar for one week at 28°C. A single colony of culture was transferred into a 500 ml Erlenmeyer flask containing 100 ml of NL200 medium consisting of mannitol 20g (Merck, Germany), cornsteep powder 20g (Marcor, Hartage ingredients, Hamburg) in 1 l tap water, and the pH was adjusted to 7.5 before sterilization, and they were placed in anorbital shaker at 28°C and cultivated at 120 rpm for 3 days. The fermentor (10 l) was inoculated with 500 ml (5%)of the 3-day-old pre-culture. Large-scale fermentation was carried out for 10 days with an aeration rate of 0.5 vvm (volume air / volume bioreactor/min) and agitation of 200 rpm (28).

The culture obtained was mixed with 2% of a filter aid (Hyphlo Supercel®, Johns Manville, USA|),and the mycelium was separated from the fermentation medium by vacuum filtration. The aqueous phase was adjusted to pH 5 (1N HCl) and extracted 3 times with the same volume of ethyl acetate, found as the best extraction solvent for non-polyenic antifungal agents. The recovered organic phase was concentrated *in vacuo* to an oily raw product (5g).

***Purification and chemical characterization of bioactive substances***

2g of active dry extracts were dissolved into 5 ml of methanol (100%), and chromatographed on a column of RP-HPLC-C18 (40g, 50µm, flow rate: 20 ml/min, Merck, Germany) and eluted with methanol 100%. This first fractionation step resulted in five fractions which were analyzed by analytical HPLC-DAD (Agilent Technologies, USA) and evaluated usingan inhouse database (29) and tested for antifungal activity.

Fraction F5 showed the strongest fungicidal activity. This fraction (137mg) was further fractionated using a column of Sephadex LH-20 (90 x 2.5 cm, flow rate: 0.5 ml/min) and developed overnight with methanol100%. Two fractions were thus separated, and the fraction FS which exhibitedthe desired activity was subjected to further purification. Final purification of the active compound has been achieved by preparative HPLC-C18 (Luna 5µm, 100A, 250*10 mm; flow rate: 4.5 ml/min) (Acetonitril (A) / H_2_O (B): 50% (A) 100% (A) in 20 min; 100% (A) from 20.1 min to 25 min) to yield the active pure compound (1.1 mg). The physicochemical properties of the compound were determined using high resolution mass spectrometry(HRMS) and liquid chromatography–mass spectrometry (LC-MS) analysis.

## Results

**Screening for non- polyenic antifungal substances**

**Fig. 1:**
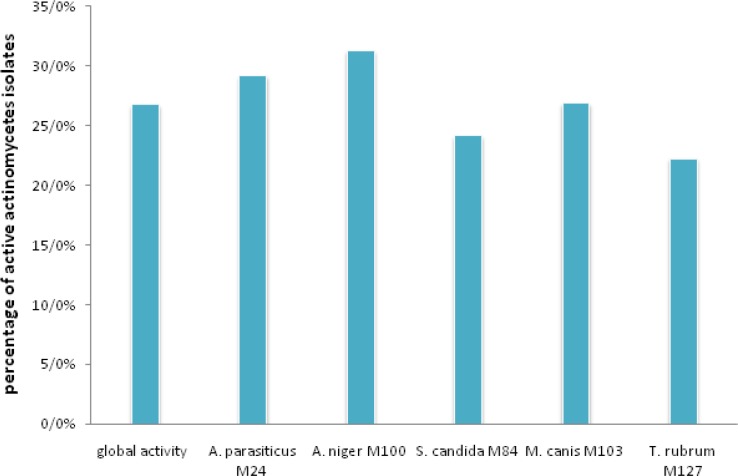
Percentage of global and specific antifungal activities

**Fig. 2:**
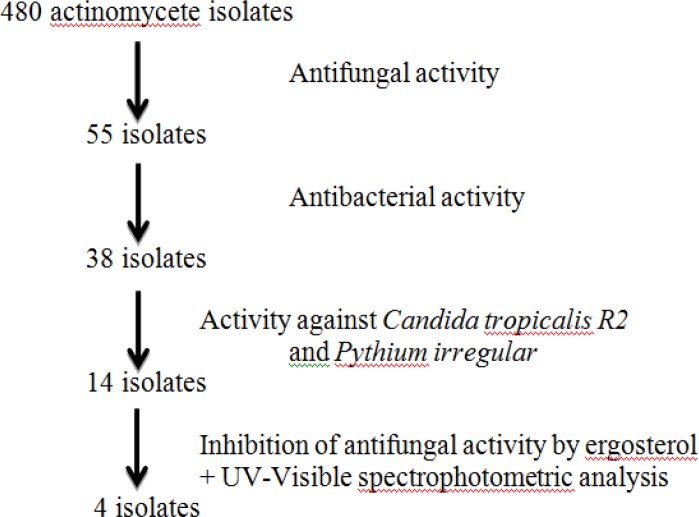
Screening steps for non-polyenic antifungal compounds producing isolates

In this study, the total number of 480 actinomycete isolates was screened on Sabouraud agar medium, and a broad-spectrum of antibacterial and antifungal activities were observed. We found that 26.8 % of the isolates were active against at least one of the test fungi. The highest percentage of activity was noted against *A. parasiticus*M24 and* A. niger *M100 with a percentage higher than 29%, but 24.2%, 26.9% and 22.3% for *S. candida M84*, *M. canis*M103, and *T. rubrum*M127, respectively. So, the latter is the most resistant of the filamentous fungi used (figure 1). The antifungal activity was observed in 55 isolates, but only 38 isolates exhibited antibacterial activity against at least one of the tested bacteria (figure 2).

The production of non-polyenic antifungal substances by the active isolates showing antifungal and antibacterial activities was investigated by assessing theiractivity against *Candida tropicalis R2* and *Pythium irregular* (resistant against amphotericin B and nystatin), ergosterol inhibition analysis, and evaluating the UV- visible spectra of active extracts. According to the data in table 2 and 3, 4 selected actinomycete isolates showed high antifungal and antibacterial activities. The isolates were more active against Gram-positive bacteria than Gram-negative bacteria. Also, they exhibited a high activity against *Candida tropicalis R2* and *Pythium irregular *(figure2).

Ergosterol present in fungal cell membrane has a very high affinity towards polyene antibiotics. Polyene drugs form complexes with ergosterol, forming channels in the fungal membrane that cause leakage of critical intracellular constituents and subsequent cell death. This behavior is exploited in a detection method developed to identify the presence of polyene class antifungals. Hence, variations in the inhibition diameters in the presence and absence of exogenous ergosterol in the culture medium indicates the implication of sterols as target of these active substances.

Table 3 shows that the isolates 1 (AS25) and 2 do not show any differences in the inhibition zones with and without ergosterol in the medium. However, isolates 3 and 4 hada reduced inhibition zone in the presence of ergosterol. The UV-visible spectra of the ethyl acetate extracts of selected active fermented strains were analyzed. None of the spectra featured the characteristic absorption bands of polyenes (series of maximum absorption between 260 and 405 nm). Isolates 3 and 4 were active against Gram positive bacteria, *Candida tropicalis *R2 and *Pythium irregular*. Their UV- visible spectra did not show the characteristic polyene peaks (30), but they hadreduced inhibition zones with ergosterol.

**Table 2:** Antifungal activity of selected actinomycetes isolates against some pathogenic filamentous fungi and polyenic resistant *Candida tropicalis R2*

Reference of isolates	Antifungal activity (in mm) against						
	*Aspergillus parasiticus M24*	*Aspergillus niger M100*	*Scorpulariopsis candida M84*	*Microsporum canis M103*	*Trichophyton rubrum M127*	*Candida tropicalis R2 *	
**Isolate 1** *		15	12	13	15	15
**Isolate 2** *	17	15	14	13	15	14
**Isolate 3** *	15	13	13	13	14	14
**Isolate 4** *	12	15	16	15	17	13

* Reference number in the collection of Actinomycetes of the Laboratory of Biology and Biotechnology of Microorganisms, Semlalia Faculty of Sciences, Marrakesh, Cadi Ayyad University, Morocco”.

**Table 3:** Results of screening program step for potential non-polyenic antifungal actinomycete isolates

Isolates	Antibacterial activity					Ergosterol effect **		UV-visible spectrum
	*St. aureus*	*M. luteus*	*B. subtilis*	*P. aeruginosa*	*E. coli*	without	with	
**Isolate 1** *	25	25	20			15	15	NP
**Isolate 2** *	21	18	18			15	15	NP
**Isolate 3** *	20	18	2			15	13	NP
**Isolate 4** *	21	18	25			17	15	NP
**Reference** ***	ND	ND	ND	ND	ND	16.0	10.0	P

* Reference number in the collection of Actinomycetes of the “Laboratory of biology and Biotechnology of microorganisms, Semlalia Faculty of Sciences, Marrakesh, Cadi Ayyad University Morocco”;

** Inhibition zones in mm with or without ergosterol at 50 mg/ml in Sabouraud medium;

*** Amphotericin B at 20 µg/well or disc; NP: non-polyenic; P: polyenic ; ND: not determined.

The selected actinomycete strain AS25, isolated from rhizospheric soil of *Alyssum spinosum* in the region of Marrakech – Morocco, attracted our attention on the basis of the strong non-polyenic antifungal activity against all tested microorganisms. Therefore, we decided to identify it using the morphological, physiological and molecular characterization methods, and to purify the active substances produced to determine its chemical structure.

**Taxonomic identification of AS25 isolate**

The AS25 strain showed a good growth and abundant mycelia on all media used after 14 days of incubation at 30°C. The aerial mycelium was grayish white on Bennett and white on ISP1, ISP2, ISP3, ISP4, and ISP5. The substrate mycelium was yellowish white on ISP1 and Bennett, Brown on ISP2 and ISP5, but Grey on ISP3 and ISP4. Diffusible and melanoid pigments were not produced on ISP7 and ISP6 (Table 4).

The physiological and biochemical characteristics of the AS25 isolate were investigated following well-established protocols and criteria described in the Bergey’s Manual of Systematic Bacteriology (31). The findings indicated that the isolate AS25 was filamentous, aerobic, Gram-positive, and a non-motile actinomycete. The carbohydrate and nitrogen source utilization profile of the isolate was also investigated using the classic methods. The results indicated that the microorganism could utilize arabinose, glucose, mannitol, serine, tyrosine, 

leucine and arginine, but not rhamnose and lactose. All data obtained with regard to the physiological and biochemical properties of the isolate, therefore, strongly confirmed that the strain belonged to the genus *Streptomyces* (Table 5).

**Table 4:** Culture and phenotypic characteristics of *Streptomyces* sp. AS25 on different ISP media after 2 weeks of incubation at 30°C

Medium	Growth *	Aerial mycelium	subsrtate mycelium
Tryptone-yeast extract agar (ISP1)	+++	white	yellowish white
Yeast extract-malt extract agar(ISP2)	+++	white	Brown
Oatmeal agar(ISP3)	+++	white	Grey
Inorganic salt-starch agar(ISP4)	+++	white	Grey
Glycerol asparagine agar (ISP5)	+++	white	Brown
Bennett	+++	grayish white	yellowish white

* : +++ Abundant growth.

**Table 5:** Physiological properties of *Streptomyces* sp. AS25 and *S. albidoflavus *using different criteria

Characteristic	*Streptomyces sp. AS25*	*S. albidoflavus*
Growth at 27°C – 40°C	+	+
Growth at pH 8.0	+	+
Utilisation of :		
Arabinose	+	+
Glucose	+	+
Rhamnose	-	-
Mannitol	+	+
Lactose	-	-
Serine	+	ND
Tyrosine	+	ND
Leucine	+	ND
Arginine	+	ND

+: positive; -: negative; ND: not determined.

**Fig. 3:**
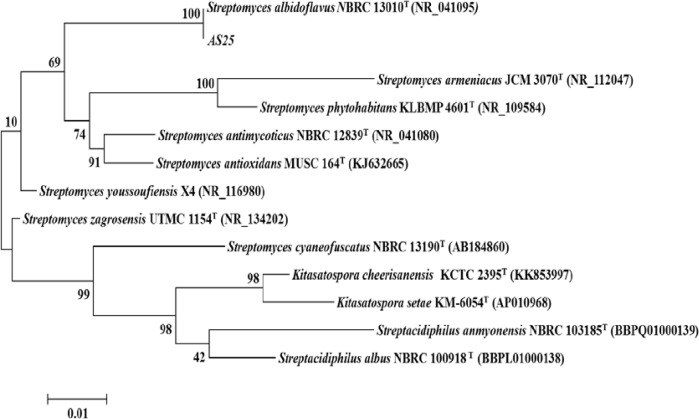
Maximum-likelihood tree based on 16S rRNA gene sequence showing the relations between strain AS25 and type species of the genus Streptomyces. The numbers at the nodes indicate the levels of bootstrap support based on maximum-likelihood analyses of 1000 resampled data sets (only values >50% are shown). Bar, 0.01 nt substitution per nt position

In order to further support the findings related to the identification of AS25isolate, the 16S rRNA gene sequence obtained was submitted to GenBank BLAST search analysis. Likewise, the results showed strong homology with those of several cultivated strains of *Streptomyces*, reaching a maximum sequence identity higher than 99%. Phylogenetic trees were then constructed using the MEGAsoftware. The nearest *Streptomyces* strain identified by the BLAST analysis was the *S. albidoflavus* strain NBRC13010^T ^with 100% of similarity. The data wasconfirmed using EzTaxon (figure 3).

**Dereplication of the chemical structure of the antifungal compound**

**Fig. 4:**
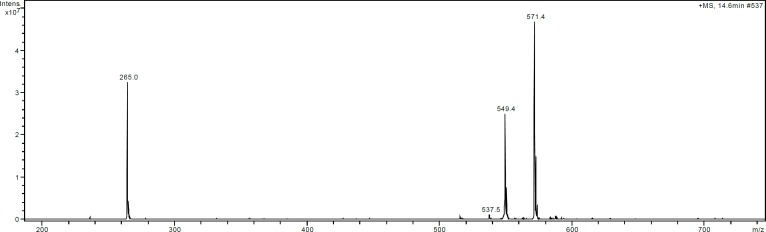
ESI-mass spectrum of the compound (positive mode; m/z 571.4 [M+Na]+).

**Fig. 5:**
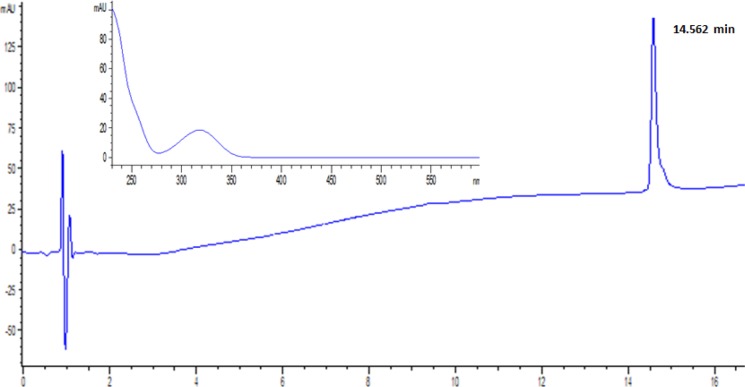
HPLC analysis of the active compound at 230 nm.Inset: UV-visible spectrum of the compound

The antifungal compound was obtained as colorless crystals and analyzed by HRMS and LC-MS (tr 14.6 min). The UV-visible and mass spectra are shown in figures 4 and 5. Analysis by electrospray ionization-mass spectrometry (ESI-MS) yielded a quasi-molecular ion peak at m*/z* 571,4 [M+Na]^+^. The compound is soluble in CHCl_3_, EtOAc, and methanol, but insoluble in water or hexane. The data obtained were evaluated using AntiBase and Dictionary of Natural Products databases (Edition 2015), and allowed the dereplication of the compound as an antimycin derivative, most likely antimycin A19 (figure 6). The minimum inhibitory concentration (MIC) of the purified molecule using *Aspergillus niger* as test microorganism was 18 µg/ml.

The basic skeleton of antimycin A19 is composed of a benzylic ring (A), a dilactone ring (B) with nine-members, and two side chains. The dihedral angle between cycles A and B was found to be 48.61° (32). This was the first antimycin with an explicit absolute configuration of chiral carbons in the acyl side chains (33). The UV-visible data showed amaximum absorptionat 228 and 321 nm, indicating the presence of the hydroxyl (or amine) and ester function in its chemical structure (34) (figure 5). The antimycins A have practically identical UV absorption spectra and also a common chromophore which is the aromatic part of the molecule (35).

## Discussion

Fungal diseases are a major public health problem today. This is because of the small number of molecules with effective activities, and alsothe emergence of resistant strains to the polyenic antifungals. In order to assess whether actinomycetesisolated from Moroccan ecosystems may be a potential source for natural bioactive compounds, the antimicrobial activity of the isolates was determined. The results show differences in the percentage of the antibacterial and especially of the antifungal activity. In previous studies, it has shown that the isolation rate of actinomycetes with antimicrobial activity is higher than 40% (16) and in others less than 10 % (36). These results confirm that the actinomycetes are able to produce a wide variety of compounds with antifungal antibiotic activity (37).

After screening for non-polyenic antifungals, we noticed that only four actinomycete isolates met the required criteria including the strain AS25 (isolate1). The results alsoshowedthat two isolates (3 and 4) have reduced antifungal activity in the presence of ergosterol. This can be explained by a coproduction of both polyene and non-polyene substances by the same strain at the same time (16).

Comparing the morphologic and physiologic properties with the representative species of the actinomycetes genus, the selected isolate AS25 was assigned to the *Streptomyces* genus. The phylogenetic analysis using the 16S rDNA sequence confirmed that it has 100% similarity with *S. albidoflavus* strain NBRC13010^T^. Stackebrandt and Ebers (38) suggested that values between 97 and 99% of similarity threshold differ between species, provided that these data are confirmed in marked phenotypic differences. However, if the percentage of similarity is higher than 97%, the DNA-DNA hybridization is required with the closest species to clarify the taxonomic position of the isolate.

Accordingto HRMS and LC-MS data, the purified compound was an antimycin derivative, and especially the antimycin A19. The MIC determined for this compound did not show a significant difference with that reported byXu et al. (33) who found a MIC of 20 μg/ml against the same species.Our data indicate for the first time the production of antimycin A19 by *Streptomyces albidoflavus*. However, antimycins in general are widespread metabolites in the genus *Streptomyces *(39).Yan et al.(40) purified antimycin A18 from an endophytic *Streptomyces albidoflavus I07A-01824* which was isolated from the leaf of *B. gymnorrhiza* collected in Shankou, Guangxi Province, China. In the same way,Xu et al. (33) identified two new antimycin derivatives A19 and A20 from the fermentation broth of *Streptomyces antibioticus H74-18,* isolated from the bottom soil of the mangrove zone in the south China Sea.

The antimycins are recognized as specific inhibitors of respiratory chain of mitochondria at the level of complex III. They are the inhibitors of the electron transfer of the ubiquinol-cytochrome c oxidoreductase (34). They have another function that directly inhibits the activity of Bcl-2-related proteins, particularly Bcl-x_L_, which is an important regulator of cell survival (41). Hockenbery et al. (42) synthesized a series of antimycin derivatives as bioactive inhibitors for Bcl-2 family proteins in an attempt to treat diseases associated with apoptosis.

The antimycins are a group of about 30 chemically closely related lactone antibiotics. The main differences between the antimycin derivatives lie in the nature of their alkyl residue at C-7 and the oxygen substituent at C-8: Acylation of the 8-hydroxy group modulates the strong antifungal, antiviral and antitumor activities (43).

The acylation of the 8-hydroxy group of antimycins showed a close relationship with their antifungal activities. Similarly, the free hydroxyl group at C8 in kitamycins a and b, as well as in urauchimycins a and b gave low antifungal activity (44). Hosotani et al. (34) reported that there are inverse relationships between antifungal activity and the length of the 7-alkyl and 8-O-acyl side chains of the antimycins.

In conclusion, our results revealed that the Moroccan ecosystems are potential sources for wide spectrum antifungal metabolite producing actinomycetes,and therefore the strain AS25 can be considered as a potential new source of effective, biologically active compound,and the screening program used to select the non-polyenic antifungals was validated based on the structural elucidation of the purified molecule.
